# Integration of Ayurveda in managing chemotherapy-induced hand–foot–skin reaction—an exploratory quasi experimental study

**DOI:** 10.3389/fmed.2025.1664832

**Published:** 2025-11-11

**Authors:** Saurav Sharma, Aiswarya Somaletha, Sreejith Kartha, Nagarajan Chockan, Pavithran Keechilat, Devipriya Soman

**Affiliations:** 1 Department of Kayachikitsa, Amrita School of Ayurveda, Amrita Vishwa Vidyapeetham (University), Kollam, Kerala, India; 2 Department of Medical Oncology & Haematology, Amrita Institute of Medical Sciences and Research Centre, Amrita Vishwa Vidyapeetham (University), Kochi, Kerala, India

**Keywords:** Ayurveda, *pariseka*, *Yastimadhu Kashaya*, *lepana*, *satadhauta ghrita*, hand–foot–skin reaction

## Abstract

**Introduction:**

Hand–foot–skin reaction (HFSR) is one of the major mucocutaneous adverse events of multi-kinase inhibitors (MKIs). Its overall incidence is 35.0%. It negatively impacts the quality of life of the patient and compliance with chemotherapy. Present management of HFSR is largely anecdotal, and the common treatment is dose regulation or discontinuation of chemotherapy. In Ayurveda, the clinical presentation of HFSR may be understood as *pitta rakta-pradhana agantuja vraṇa* in *hasta* and *pada* caused by MKIs. The present study aimed to evaluate the Ayurveda treatment protocol as an add-on to the standard of care.

**Methodology:**

This is a double-arm-controlled interventional study with a quasi-experimental design, registered in the Clinical Trial Registry of India under CTRI/2022/03/040929, with 22 participants from the Medical Oncology Department and Integrative Medicine, Amrita Institute of Medical Sciences, Kochi. The study group received an add-on treatment to the standard of care (SOC). This included *Yasṭimadhu Kashaya pariseka* (from days 1 to 30), followed by *Satadhauta ghrita Lepana* (from days 6 to 30) twice daily immediately after *pariseka*. A gap of 1 h was maintained between the Ayurveda treatment and SOC as prescribed by the oncologist.

**Results and conclusion:**

The study group demonstrated a significant reduction in pain (VAS) and improvement in quality of life (HF-QoL) compared to the control group. *Yasṭimadhu* is vrana *sandhaneeya, varnya, daha prashamana, shothahara,* and *shonitasthapana. Ghrita* is *Vata pittahara, sheeta veerya,* and *dahaprashamana,* and it facilitates *ropana*. HF-QoL improved due to effective management of symptoms. The pilot study suggested that an integrated therapy is feasible in HFSR patients, which can possibly support improving the quality of life for cancer patients undergoing chemotherapy.

**Clinical trial registration:**

CTRI/2022/03/040929, https://www.ctri.nic.in/Clinicaltrials/pmaindet2.php?EncHid=NjMwMTE=&Enc=&userName=.

## 1 Introduction

Multi-kinase inhibitors (MKIs) are targeted chemotherapy agents that have proven to be clinically effective in the treatment of a wide range of cancers (1). However, they are associated with many adverse events, and the major mucocutaneous adverse event of MKIs is the hand–foot–skin reaction (HFSR) (2). HFSR affects the skin on the palmar and plantar surfaces. Beginning as a mild skin reaction, the condition can quickly progress into a painful, debilitating condition. It affects the person’s ability to perform activities and has an undesirable influence on the quality of life of the patient and also compliance with cancer therapy (2). Present management of HFSR is largely anecdotal, and the most common method of treatment for grade 2 and grade 3 HFSR is dose regulation or discontinuation of chemotherapy (3). The cessation of chemotherapy because of unbearable symptoms of HFSR could increase the mortality rate of cancer patients. While analyzing the effect of MKIs on the body, they seem to possess *guna* similar to those described in Ayurveda treatises as *visa guna* (attributes of poison), such as *tiksna* (sharply acting), *asu* (rapid acting)*, vyavayi* (which pervades the whole body before getting digested)*, suksma* (subtleness)*, usna* (hot), *and ruksa* (dryness) (4). These attributes cause *pitta rakta pradhana tridosha dusti* (ulcer with clinical presentation of three *doshas* with predominance of *pitta* and *rakta*) in the body (5). In Ayurveda, the clinical presentation of HFSR may be understood as *pitta rakta pradhana agantuja vrana* (exogenous ulcer) in *hasta* and *pada* (hand and foot) caused due to *tiksnadi gunas* (attributes of poison, such as sharply acting) of MKIs. The first line of management of *agantuja vrana* is *pittavat sita kriya* (6) (medicines and therapies that are cold, intended to pacify *pitta*). In order to manage *pitta rakta pradhana dusti* (clinical presentation of impairment of *rakta*)*, bahya prayogas* (topical applications) that are *sita* (cold) in *guna* are indicated (7). Evidences suggest that *Glycyrrhiza glabra* (*Yashtimadhu*) and ghee have anti-inflammatory activity and wound-healing potential (8). Wayal et al. reported that the process of *satadhauta* or washing ghee with water 100 times, increases the moisture content in ghee, which may be useful for skin hydration and cooling while used topically (9). The present study aims to evaluate the feasibility of an *Ayurveda* treatment protocol containing *pariseka* or wash, with *Yastimadhu* (*Glycyrrhiza glabra*) *Kashaya,* followed by *lepana* (anointment) with *satadhauta ghrita* as add-on therapy in reducing the symptoms of HFSR due to MKIs.

## 2 Methods

### 2.1 Study design and ethical considerations

**Figure 1:**
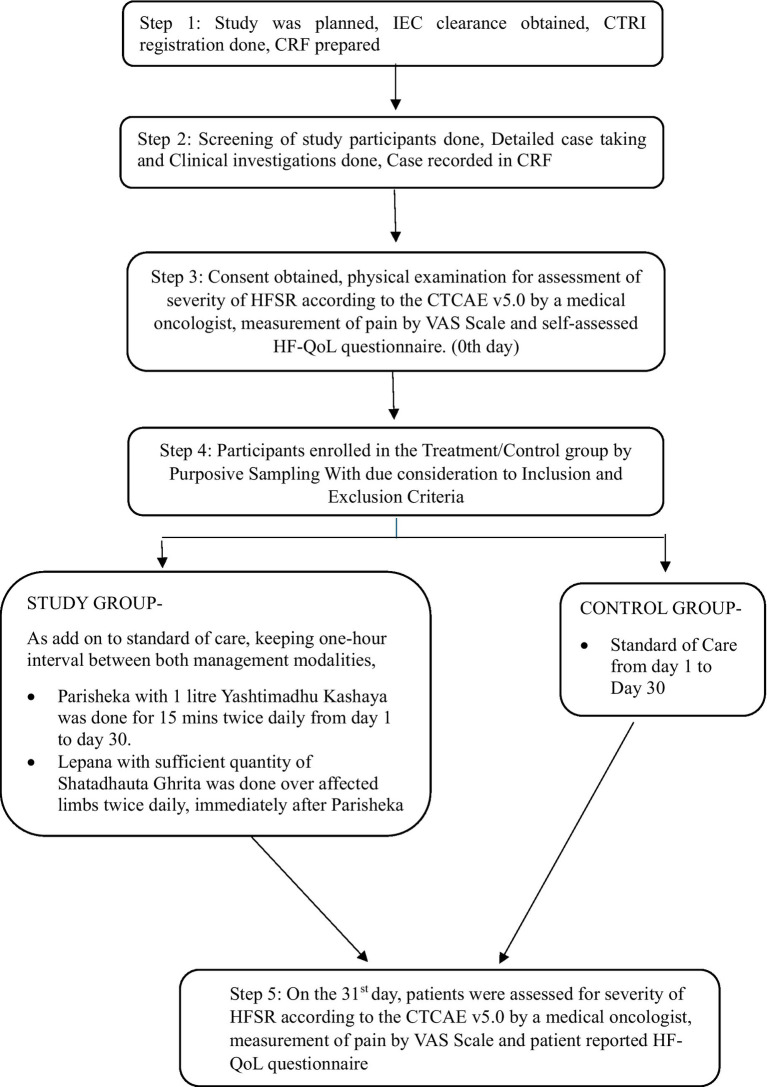
Methodology.

This is a double-arm, interventional study with a control. Participants were selected from the OPD and IPD of Medical Oncology and the Department of Integrative Medicine, Amrita Institute of Medical Sciences, Kochi. The trial was conducted between March 2022 and September 2022, and a diagrammatic presentation of the patient flow is provided (Figure 1). The Clinical Research Act (enacted 14 April 2017) and the amended October 2013 Declaration of Helsinki have been followed in this investigation. The study was approved by the Institutional Ethics Committee of Amrita Institute of Medical Sciences under IEC-AIMS-2021-AYUR-068A. Furthermore, all relevant regional, national, and international laws have been followed. This study is registered in the Clinical Trial Registry of India under CTRI/2022/03/040929.

### 2.2 Participant selection

Participants who had undergone chemotherapy with MKIs and were consequently diagnosed with HFSR by a medical oncologist, based on CTCAE v5.0 criteria, were screened and selected based on the inclusion and exclusion criteria.

#### 2.2.1 Inclusion criteria

- Age: 18 years–60 years.
- Diagnosed with a malignant tumor with pathological or cytological findings as evidence.
- An Eastern Cooperative Oncology Group performance status ≤3.
- Treatment with MKIs and consequent occurrence of hand–foot–skin reaction of any grade.
- Life expectancy >3 months.
- Those who are willing to participate in this study and have signed the informed consent forms.

To confirm the presence of HFSR in patients, the CTCAE v5.0 grading system was utilized (10). Although there is no specific grading for HFSR within this system, it is noted that the grading criteria for HFS (palmar-plantar erythrodysesthesia) are applicable and can be used equivalently as follows:

##### 2.2.1.1 Grade 1

Minimal skin changes or dermatitis (e.g., erythema, edema, or hyperkeratosis) without pain.

##### 2.2.1.2 Grade 2

Skin changes (e.g., peeling, blisters, bleeding, fissures, edema, or hyperkeratosis) with pain, limiting instrumental ADL.

##### 2.2.1.3 Grade 3

Severe skin changes (e.g., peeling, blisters, bleeding, fissures, edema, or hyperkeratosis) with pain, limiting self-care ADL.

#### 2.2.2 Exclusion criteria

- Dermatological toxicities were not induced by MKIs.
- Concurrent acne vulgaris, eczema, psoriasis, and other skin diseases.
- Abnormal cognition and speech causing difficulty with comprehension, expression, and obtaining informed consent.

### 2.3 Enrolment and intervention

The eligible participants were explained regarding SOC and the add-on traditional therapies and further left to his/her choice to select either the SOC alone or the same with add-on traditional therapies, including *pariseka* and *lepa*. Henceforth, they were assigned to either the control or study group as per their choice.

In both the control and study groups, the standard of care (SOC) was prescribed as per the respective grade of HFSR. In grade 1 HFSR, the primary interventions include the application of moisturizing creams and topical keratolytics such as urea and salicylic acid creams. Additionally, cushioning of the affected areas is recommended, and no dose reduction of chemotherapy. For grade 2 HFSR, the same symptomatic measures as in grade 1 are used, and the treatment is enhanced by the application of potent corticosteroids and analgesics to inflammatory lesions. In this grade, a dose reduction or interruption of chemotherapy may be considered. In cases of grade 3 HFSR, the interventions from grade 2 are continued, with the addition of antiseptic treatment for blisters and erosions. Clinical management may include dose reduction, re-escalation, or even the interruption or discontinuation of chemotherapy, depending on clinical judgment and patient preferences (11, 12).

The study group received an add-on treatment to the SOC. This included *pariseka* with *Yastimadhu Kashaya* (1 liter at room temperature for each administration). The participants had to prepare *kashaya* for *pariseka* as per the instructions given. A measure of *62*.5 g of coarse *Yastimadhu Churna* (powder) had to be boiled in 4 liters of water, reduced to 2 liters, and left to cool to room temperature. It was then divided into two equal portions to be used in the morning and evening for *pariseka,* or washed over the affected area for 15 min twice daily from days 1 to 30, followed by *lepana* with *shatadhauta ghrita* (QS) twice daily immediately after *pariseka* from days 6 to 30. An interval of 1 h was maintained between the Ayurveda treatment and SOC as prescribed by an oncologist.

### 2.4 Outcome measures

The principal objective was the evaluation of alterations in HFSR grades from baseline to the 30-day mark, using the CTCAE v5.0 criteria; evaluation of variations in pain severity, gauged through the VAS score; and analysis of quality of life utilizing the HF-QoL questionnaire (13).

### 2.5 Sample size

Being an exploratory study using non-probability sampling, a minimum sample size of 30 was planned for the 6-month trial period. However, due to the constraints of the recruitment timeline, only 23 participants were ultimately enrolled in the study.

### 2.6 Trial drugs

Both *Satadhauta ghrita* (Batch No.: #8281) and *Yashtimadhu Churna* were procured from A V Oushadhasala, Thattarkonam, Kollam, Kerala – 691005, a GMP-certified company. The drugs were stored in airtight polythene containers arranged in almirahs.

### 2.7 Statistical analyses

The effect of the intervention within the group was assessed using a paired *t*-test, while the between-group effect was assessed using an independent *t*-test. Descriptive statistics were used for demographic variable analysis using IBM SPSS version 20. The significance level was set at a *p*-value of <0.05, considered statistically significant, and a *p*-value of <0.001 was regarded as highly significant. Results with a *p*-value of >0.05 were deemed insignificant.

## 3 Results

**Figure 2:**
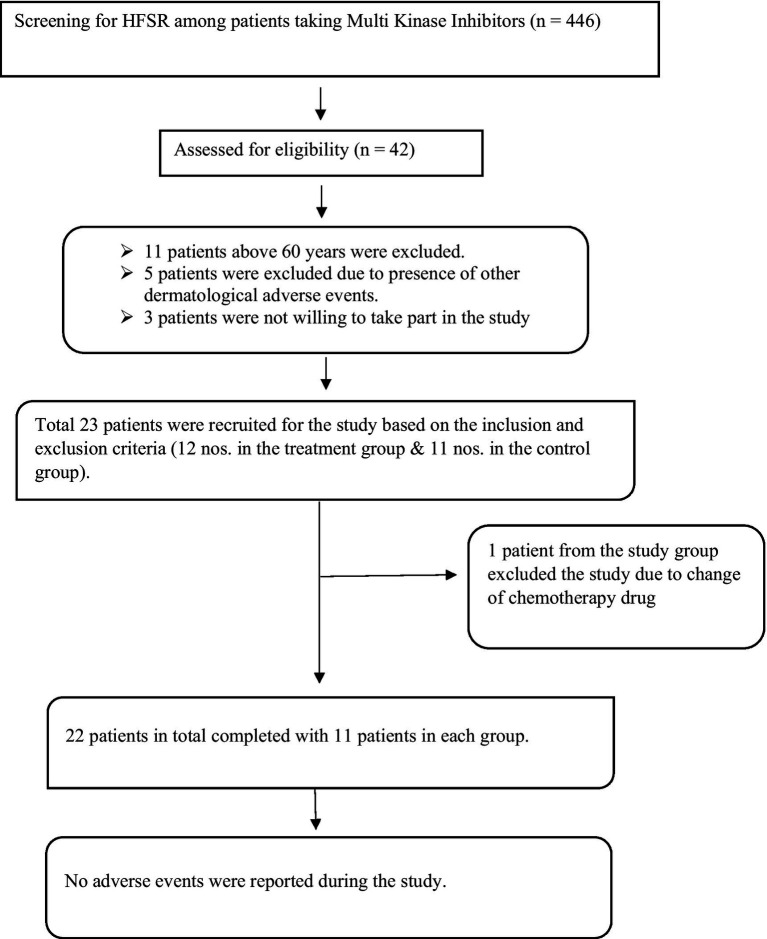
Patients’ inflow chart.

The patients who were undergoing chemotherapy via MKIs were screened based on inclusion and exclusion criteria. Informed consent was obtained from 23 patients. During the study period, one participant was excluded from the study. She was a known case of clear cell carcinoma with metastasis. She was under treatment with pazopanib and presented with grade 2 HFSR. She followed the Ayurveda treatment protocol till day 13. After the 13th day, she discontinued the treatment for HFSR because her chemotherapy drug was changed to immunotherapy due to the progression of cancer (Figure 2).

### 3.1 Patient demographics and clinical characteristics of the HFSR population

The demographic data and clinical characteristics of the population experiencing HFSR are summarized in Table 1. Both the study and control groups were tested for homogeneity and were found to be homogeneous with respect to baseline data. The mean age of patients was 55.9 years in the treatment group and 50.8 years in the control group. In both groups, the female population exceeded the male population by 9%.

**Table 1:** Baseline data.

Characteristics	Treatment group	Control group	
Age	55.9 (±2.7)	50.8 (±9.4)	
35–40	0 (0%)	2 (18.2%)	
40–45	0 (0%)	1 (9.1%)	
50–55	5 (45.5%)	3 (27.3%)	
55–60	6 (54.5%)	5 (45.4%)	
Gender			
Female	6 (54.5%)	6 (54.5%)	
Male	5 (45.5%)	5 (45.5%)	
Site of cancer			
Appendix	1 (9.1%)	0 (0%)	
Colon	0 (0%)	1 (9.1%)	
Kidney	1 (9.1%)	1 (9.1%)	
Liver	4 (36.3%)	5 (45.4%)	
Lung	0 (0%)	1 (9.1%)	
Ovary	2 (18.2%)	1 (9.1%)	
Rectum	1 (9.1%)	0 (0%)	
Soft tissue	0 (0%)	1 (9.1%)	
Thyroid	0 (0%)	1 (9.1%)	
Urinary bladder	1 (9.1%)	0 (0%)	
Uterus	1 (9.1%)	0 (0%)	
Chemotherapy drug			
Gefitinib	0 (0%)	1 (9.1%)	
Lenvatinib	2 (18.2%)	0 (0%)	
Pazopanib	4 (36.3%)	2 (18.2%)	
Regorafenib	3 (27.3%)	2 (18.2%)	
Sorafenib	2 (18.2%)	6 (54.5%)	
HFSR on hands	3 (27.3%)	1 (9.1%)	
HFSR on foot	10 (90.9%)	10 (90.9%)	
HFSR grade	1.9 (±0.09)	1.8 (±0.12)	*p* = 0.56
Quality of life	40 (±22.6)	26.6 (±18.8)	*p* = 0.15
VAS scale	6.7 (±2.7)	5.8 (±3.2)	*p* = 0.48

The primary site of cancer was the liver, i.e., 45.4% in the control group and 36.3% in the treatment group. A smaller proportion of participants in each group were diagnosed with cancer at other sites, including the appendix, colon, kidney, lung, rectum, soft tissue, thyroid, urinary bladder, and uterus. It is observed that half of the participants in the treatment group and 18% in the control group received sorafenib. Additionally, 36.3% of the treatment group and 18% of the control group received pazopanib. Treatment with regorafenib was observed in 27% of the treatment group and 18% of the control group, while only one participant in the control group received gefitinib. HFSR predominantly occurred on the feet in the majority of participants in both groups. The mean HFSR grade before treatment was 1.9 (±0.09) in the treatment group and 1.8 (±0.12) in the control group. The mean Hand-Foot Quality of Life (HFQoL) score prior to treatment was 40 (±22.6) in the treatment group, compared to 26.6 (±18.8) in the control group. Furthermore, the mean visual analog scale (VAS) score before treatment was 6.7 (±2.7) in the treatment group, whereas it was 3.2 (±3.2) in the control group.

### 3.2 Effect on the grade of HFSR

The mean grade of HFSR according to CTCAE v5.0 in the control group before intervention was 1.82 (±0.405), which remained the same after treatment. There was no progression of HFSR to a higher grade.

The mean grade of HFSR according to CTCAE v5.0 in the treatment group before intervention was 1.91 (±0.302), which remained the same after treatment. There was no progression of HFSR to a higher grade.

### 3.3 Effect on pain

**Figure 3:**
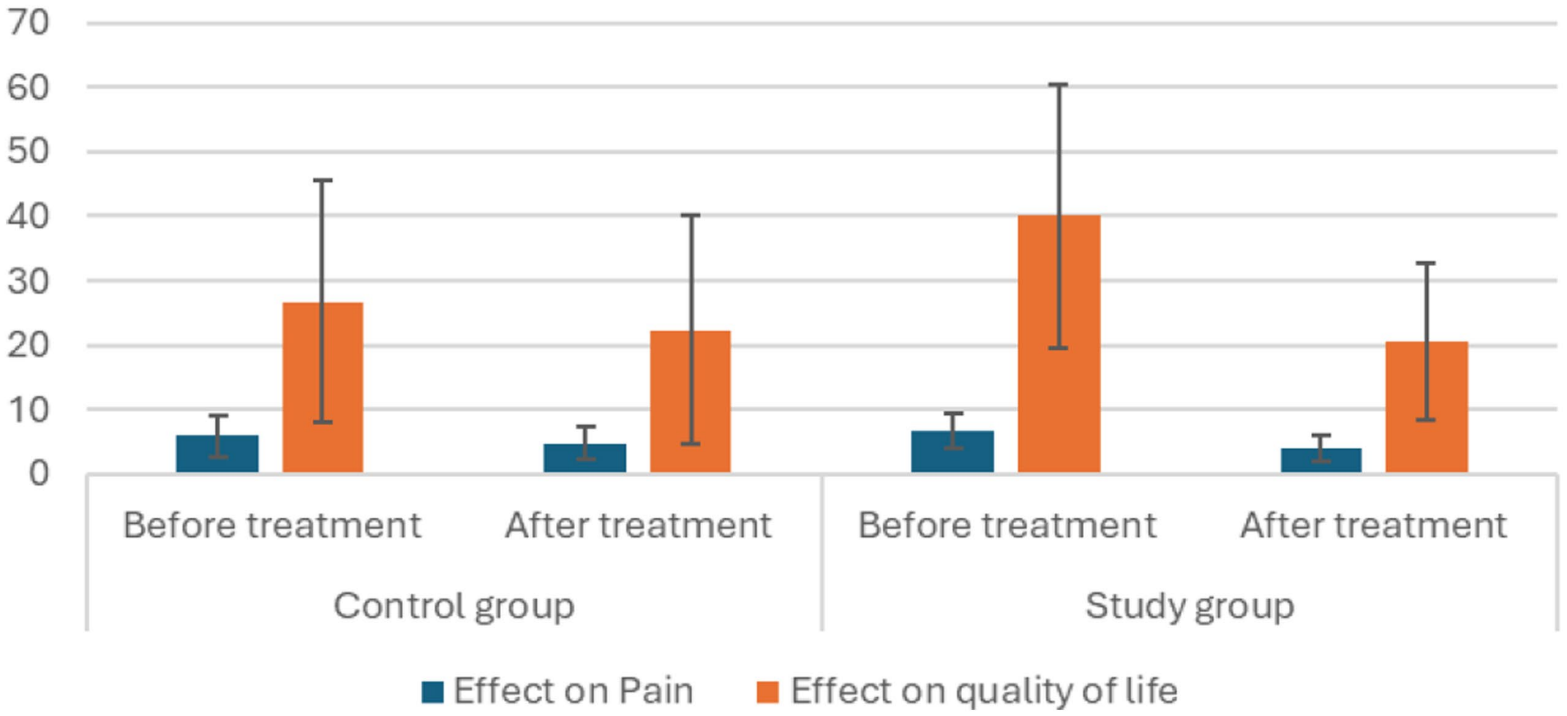
Effect of therapy on pain and QoL within the groups.

In the control group, the mean VAS score before the intervention was 5.8 (± 3.2). After the intervention, it decreased to 4.7 (±2.6), with a significant reduction of 1.09 points. The *t*-value for this change was 4.4, with a *p*-value of 0.001, indicating statistical significance (Table 2; Figure 3).

**Table 2:** Effect of therapy on pain and QOL within the groups.

Statistical parameters		Effect on pain				Effect on QOL			
		Control group		Treatment group		Control group		Treatment group	
		B.T.	A.T.	B.T.	A.T.	B.T.	A.T.	B.T.	A.T.
Total Number		11	11	11	11	11	11	11	11
Mean		5.8	4.7	6.7	4.0	26.64	22.36	40.0	22.6
Standard deviation		3.2	2.6	2.7	2.1	18.8	17.7	20.5	12.3
BT-AT Mean		1.09		2.7		4.3		19.5	
Standard deviation		o.8		1.6		3.9		12.3	
Standard error mean		0.25		0.49		1.2		3.7	
95% confidence interval	L	0.532		1.640		1.7		11.3	
	U	1.649		3.814		6.9		27.8	
*T*-value		4.4		5.6		3.7		5.3	
*P*-value		0.001		0.000		0.004		0.000	

QOL, quality of life; BT, before treatment; AT, after treatment; L, lower; U, upper.

In the treatment group, the mean VAS score before the intervention was 6.7 (±2.7), which decreased to 4.0 (±2.1) post-intervention. This represents a significant reduction of 2.7 points, with a *t*-value of 5.6 and a *p*-value of <0.001, showing a highly significant improvement (Table 2; Figure 3).

**Figure 4:**
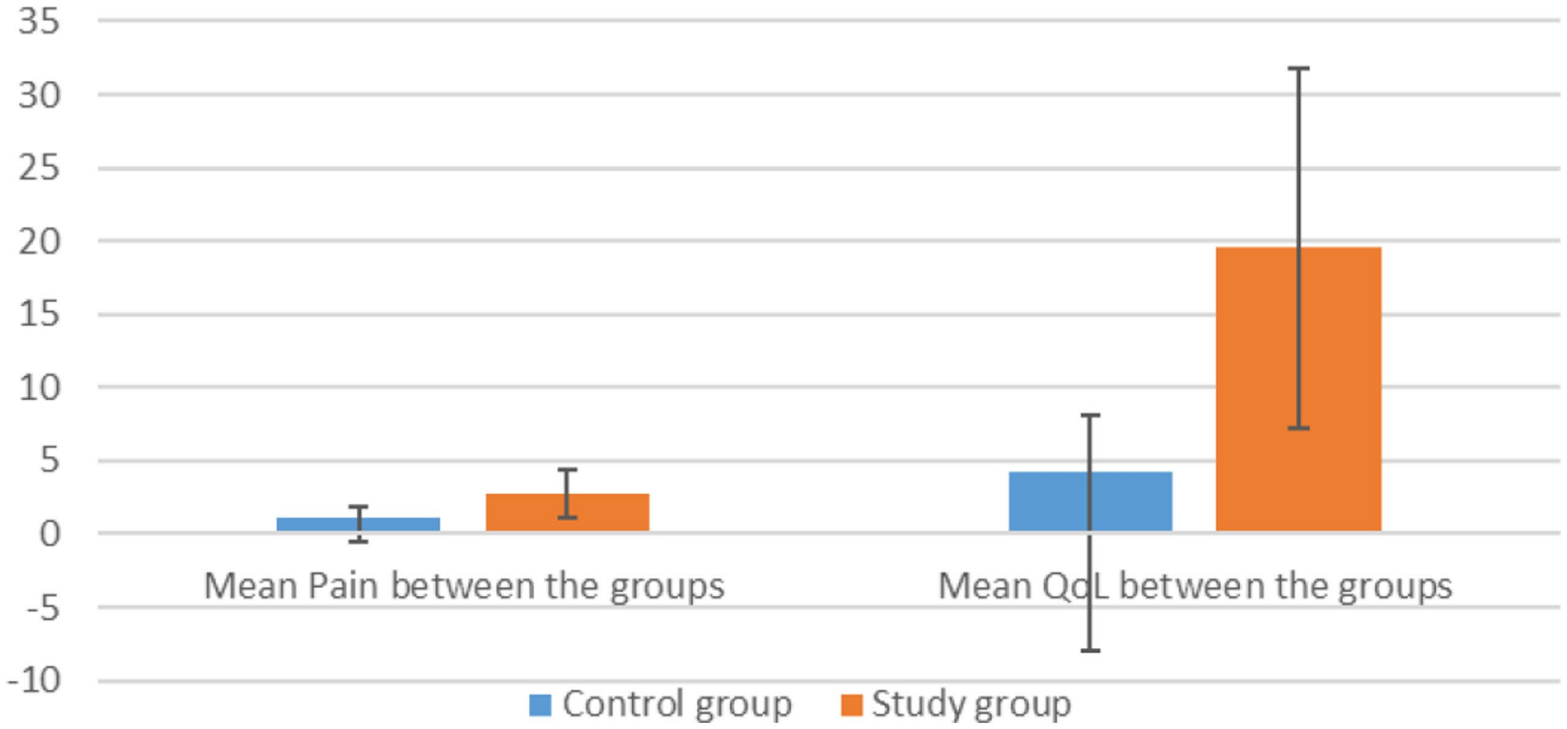
Effect of therapy on pain and QoL between the groups.

Comparing the mean differences in pain reduction between the treatment and control groups, a significant difference is noted (Figure 4). The treatment group showed a greater reduction in pain (mean difference = 2.7) compared to the control group (mean difference = 1.09). The *t*-test results indicate that this difference is statistically significant, with a *p*-value of 0.007 under the assumption of equal variances and 0.009 when variances are not assumed (Tables 3, 4). These results suggest that the treatment was more effective than the control intervention in reducing pain.

**Table 3:** Effect of therapy on pain and QOL between the groups.

Statistical parameters	Effect on pain		Effect on QOL	
	Control group	Treatment group	Control group	Treatment group
Total number	11	11	11	11
Mean	1.09	2.73	4.27	19.55
Standard deviation	0.831	1.618	3.875	12.275
Standard error mean	0.251	0.488	1.168	3.701

**Table 4:** Independent sample test.

Statistical parameters		Levene’s test for equality of variances		*t*-test for equality of means						
									95% Confidence Interval of the Difference	
		*F*	Sig.	*t*	df	Sig. (2-tailed)	Mean difference	Std. Error difference	Lower	Upper
Mean difference pain	Equal variances assumed	2.668	0.118	2.983	20	0.007	1.636	0.548	0.492	2.780
	Equal variances not assumed			2.983	14.934	0.009	1.636	0.548	0.467	2.806
Mean difference HFQoL	Equal variances assumed	15.138	0.001	3.935	20	0.001	15.273	3.881	7.177	23.369
	Equal variances not assumed			3.935	11.974	0.002	15.273	3.881	6.815	23.731

### 3.4 Effect on quality of life

In the control group, the mean HFQoL score before intervention was 26.64 (±18.8). Following intervention, it decreased to 22.36 (±17.7), indicating a significant reduction of 4.3 points. This suggested an improvement in the quality of life for individuals in the control group. The *t*-value for this change was 3.7, with a *p*-value of 0.004 (Table 2; Figure 3).

In the treatment group, the mean HFQoL score before intervention was 40 (±22.6). After intervention, it significantly decreased to 20.5 (±12.4), showing a substantial reduction of 19.5 points. This marked an improvement in the quality of life of individuals in the intervention group. The *t*-value for this change was 5.3, with a *p*-value of less than 0.001 (Table 2; Figure 3).

The treatment group demonstrated a significantly greater improvement in quality of life compared to the control group, with a mean difference of 15.273 points on the HFQoL score (Figure 4). This difference is statistically significant, with a *p*-value of less than 0.01, indicating a high level of significance in the observed improvement (Tables 3, 4).

## 4 Discussion

The treatment group demonstrated a significant relief of 40%, calculated based on the VAS Score. A significant mean difference in pain between the two groups was noted, with the treatment group experiencing better pain relief than the control group.

The inflammation associated with HFSR leads to erythema, swelling, hyperkeratotic plaques, blistering, ulceration, bleeding, and pain (14). Pain linked to inflammation typically resolves as the underlying pathology heals. Various studies have investigated the anti-inflammatory and wound-healing properties of *Glycyrrhiza glabra* (GG) and ghee.

Siriwattanasatorn et al. (15) reported that ethanolic extracts of GG could lessen the generation of superoxide anion, curtail nitric oxide production, enhance fibroblast proliferation, and hasten wound closure. The anti-inflammatory effects of glabridin, a compound in GG, have been demonstrated *in vitro* through its inhibition of superoxide anion production and cyclooxygenase activities (16). Glycyrrhizin acid has also been shown to reduce erythema, edema, and itching scores through topical application in a double-blind clinical trial (16). According to Castangia et al. (17), the high antioxidant content in the topical application of liquorice extract formulations helps counteract oxidative stress damage and maintain skin homeostasis. An experimental study on excision wounds in adult Wistar albino rats explored the wound-healing potential of aqueous extracts of GG and cow’s ghee. The wound-healing efficacy was assessed using immunohistochemical (IHC) parameters, focusing on the inflammatory response, assessed by the level of interleukin-1β (IL1β) and tissue remodeling through the activity of myofibroblasts (8).

Regarding the effect on myofibroblasts, GG exhibited anti-inflammatory and skin regeneration activities. Proliferation of dermal fibroblasts was observed. The process of dermal fibrosis was found to decelerate significantly due to glycyrrhizin, by its potential to reduce collagen content and the number of myofibroblasts (8). GG-treated wound healed better, pointing to optimal myofibroblast activity. It amplified myofibroblast activity during the early phases of healing, helping with contraction and wound closure. Fibrosis and scar formation were further absent in the process of wound healing as GG reduced myofibroblast activity, thereby decreasing excessive collagen deposition at the end of the healing phase.

Saturated fatty acids and polyunsaturated fatty acids (PUFAs), such as linolenic acid (n-3), linoleic acid (n-6), and oleic acid (n-9), are the major components of ghee (18). PUFAs, in addition to their structural role, can modulate cell–cell interaction and intracellular signal transduction (19). n-3 and n-6 PUFAs can stimulate epithelial cell proliferation *in vitro*, playing a fundamental role during healing (20). Prasad and Dorle (27) report that the topical application of ghee leads to early epithelialization, due to an increase in the volume of hydroxy proline (21). Moreover, ghee-treated wounds showed significantly higher activity of myofibroblasts initially. Later, its activity was significantly reduced. This reaffirms the role of ghee in early wound closure without scar formation (8).

The percentage of relief calculated based on the HFQoL score showed a significant relief of 48.8% in the treatment group. There was a significant difference in the mean difference in the HFQoL score between the two groups. The treatment group had a better quality of life than the control group.

The reduction in the score of HFQoL, pointing to improvement in the quality of life, is mainly due to effective management of symptoms such as reduction of swelling, redness, and pain. The relief from pain and intolerance to touch improves the participant’s ability to perform self-care and other activities, which in turn improves their psychology and social life (13).

The combined actions of the formulations and the therapy, along with the standard of care, would have contributed to an improvement in the quality of life of participants in the treatment group compared to the control group.

### 4.1 Probable mode of action of *pariseka* with *Yastimadhu Kashaya*

*Anusna pariseka* (not too hot), according to *the Dvivraneeya chikitsa* in the *Sushruta Samhita*, is *a bahya prayoga* (external application) indicated for *Pitta-Rakta-Abhighata-Visa Nimittaja Vrana* (ulcer with the clinical presentation of pitta and rakta caused due to poisoning). For *Anusna pariseka*, sweet (*madhura*) drugs, such as those of the *kakolyadi gana,* are prescribed in this context (22). *Yastimadhu* is a *madhura rasa drug* and is also *sheeta* (cooling) in nature. *Acharya Charaka* describes *Yastimadhu* as *vrana sandhaneeya* (wound healing), *varnya* (enhancing complexion), *daha prashamana* (alleviating burning sensation), and *shonitasthapana* (stopping bleeding). *Madanadi Nighantu* describes *Yaṣṭimadhu* as *vrana shodhana* (cleansing wounds), *ropana* (healing wounds), *varnakrut* (enhancing complexion), and *rakta pitta hara* (alleviating blood-related disorders). It can aid in controlling swelling (*shopha*) and creates an environment conducive to the healing (*ropana*) of wounds (*vrana*) (23).

### 4.2 Probable mode of action of *lepana* with *shatadhauta ghrita*

*Lepana* is described in the *Vranaalepanabandhanavidhi adhyaya* as pacifying doshas (*dosha shamana*), alleviating burning sensation (*dahahara*), pain-relieving (*rujahara*), soothing the skin (*tvak prasadana*), and purifying the blood (*rakta prasadana*) (24). *Ghrita* (clarified butter) is considered *Vata Pittahara* (pacifying Vata and Pitta doshas), *sheeta* in *veerya* (cooling in potency), and *dahaprashamana* (alleviating burning sensation), and it facilitates healing (*ropana*). Hema Sharma Dutta et al. suggest that the topical application of cow ghee on wounds can act as both *samshodhana* (cleansing) and *samshamana* (pacifying) (25). *Shatadhauta ghrita* is indicated in conditions such as *agni visarpa*, which involves pronounced *pitta-rakta dushti* (imbalance of Pitta and blood). In the *nibandha sangraha vyakhya*, it is recommended for external application in *pitta-rakta* predominant wounds and wounds caused by *visha* (poison) (22). Wayal et al. (9) suggested that the process of manufacturing *shatadhauta ghrita,* i.e., washing ghee in water 100 times, increases the cooling effect of the topical application (9). According to Barkin et al. (28), the nerve conduction velocity slows down on cold application to the skin, a phenomenon called cold-induced neuropraxia. This also induces an analgesic effect by reducing muscle spindle activity and causes vasoconstriction, which slows blood flow and reduces swelling (9).

## 5 Conclusion

Chemotherapy is a prevalent method for treating cancer, yet it presents notable advantages and disadvantages. The efficacy of traditional chemotherapy is often hindered by significant side effects such as hepatotoxicity, nausea, fatigue, hair loss, and hand-foot syndrome. Despite these limitations, chemotherapy remains a vital treatment option for various cancers, complementing surgery, radiotherapy, and targeted therapies. To address these challenges, researchers are investigating the potential for combined therapies incorporating herbal products and dietary supplements to improve outcomes and reduce adverse effects (26).

The addition of Ayurvedic interventions has demonstrated a beneficial effect on addressing the challenges associated with chemotherapy by reducing pain and enhancing the quality of life for patients. Although the Ayurveda add-on did not alter the severity of hand–foot–skin reaction (HFSR), its integration into chemotherapy regimens was beneficial in allowing patients to continue their treatment with reduced discomfort. This suggests that an integrated therapy is feasible in HFSR patients and can play a supportive role in improving the quality of life for cancer patients undergoing chemotherapy.

### 5.1 Limitations of the study

The study duration was 30 days per participant. Within this period, there was no change in the grade of HFSR in both groups. As the study was time-bound, long-term follow-up to assess the sustained efficacy, as well as the assessment of long-term safety of the integrative approach, was not possible. The study being an exploratory one, non-probability sampling was used, and following the rules of thumb, a small sample was adopted here.

Moreover, the study included vulnerable participants. They were in pain due to HFSR and with reduced QOL. They were left to choose the treatment modality they preferred, and hence, randomization was not possible. This sort of allocation of participants would have introduced bias into the study.

The interventions included in this study were external therapies. The introduction of sham procedures as a control would add a burden to the participants, as they are less likely to contribute to alleviating the condition in those who are already in pain. Hence, the study was taken up with only conventional management in the control group and conventional management with add-on Ayurveda topical interventions in the study group. This limited the study to being blinded.

The potential confounding factors, including patient expectation and attention effect, were not addressed in the study. As an exploratory study, our primary aim was to generate preliminary evidence. We focused on variables such as pain, HFSR grade, and QoL in the sample to establish a foundation for more focused research. Moreover, controlling these complex psychological variables was beyond our scope with an exploratory design.

### 5.2 Suggestions

A clinical trial to assess the effectiveness of the integrative approach to the prophylactic management of HFSR is recommended. More *in vivo* studies are needed to analyze the pharmacodynamics and pharmacokinetics of the drugs. Additionally, a three-arm study could be conducted: one arm receiving standard care alone, another arm receiving integrative treatment, and a third arm receiving Ayurveda care alone for managing the complications of chemotherapy.

## Data Availability

The original contributions presented in the study are included in the article/supplementary material, further inquiries can be directed to the corresponding author.
